# Notification data and criteria during a large Q-fever epidemic reassessed

**DOI:** 10.1017/S0950268819000736

**Published:** 2019-05-09

**Authors:** D. A. T. Hanssen, G. Morroy, M. M. A. de Lange, C. C. H. Wielders, W. van der Hoek, F. Dijkstra, P. M. Schneeberger

**Affiliations:** 1 Department of Medical Microbiology, Maastricht University Medical Center, The Netherlands; 2 Department of Infectious Diseases, Public Health Service, Hart voor Brabant, ‘s-Hertogenbosch, The Netherlands; 3 Centre for Infectious Disease Control, National Institute for Public Health and the Environment (RIVM), Bilthoven, The Netherlands; 4 Department of Medical Microbiology and Infection Control, Jeroen Bosch Hospital, ‘s-Hertogenbosch, The Netherlands

**Keywords:** Acute Q-fever, notification criteria, PCR, serology

## Abstract

From 2007 to 2010, the largest reported Q-fever epidemic occurred in the Netherlands with 4026 notified laboratory-confirmed cases. During the course of the epidemic, health-seeking behaviour changed and awareness among health professionals increased. Changes in laboratory workflows were implemented. The aim of this study was to analyse how these changes instigated adjustments of notification criteria and how these adjustments affected the monitoring and interpretation of the epidemic. We used the articles on laboratory procedures related to the epidemic and a description of the changes that were made to the notification criteria. We compared the output of a regional laboratory with notifications to the regional Public Health Service and the national register of infectious diseases. We compared the international notification criteria for acute Q-fever. Screening with ELISA IgM phase II and PCR was added to the diagnostic workflow. In the course of the epidemic, serology often revealed a positive IgG/IgM result although cases were not infected recently. With increasing background seroprevalence, the presence of IgM antibodies can only be suggestive for acute Q-fever and has to be confirmed either by seroconversion of IgG or a positive PCR result. Differences in sero-epidemiology make it unlikely that full harmonisation of notification criteria between countries is feasible.

Abbreviations: CDC, Centers for Disease Control and Prevention; CFT, complement fixation test; ECDC, European Centre for Disease Prevention and Control; ELISA, enzyme-linked immunosorbent assay; IFA, indirect immunofluorescence antibody assay; IHC, Immunohistochemistry; LMM JBZ, Laboratory of Medical microbiology of the Jeroen Bosch Hospital; PHS HvB, Public Health Service Hart voor Brabant; PCR, polymerase chain reaction; qPCR, quantitative polymerase chain reaction; RIVM, National Institute for Public Health and the Environment.

## Introduction

Q-fever is a zoonosis caused by *Coxiella burnetii* (*C. burnetii*). Humans are primarily infected by inhaling contaminated aerosols [1]. Ruminants, especially goats and sheep, are considered the primary reservoirs for human infection. Acute infection with *C. burnetii* in humans is most often (60%) asymptomatic. In symptomatic acute infections, it presents as a non-specific influenza-like illness, pneumonia or hepatitis [2]. Patients with cardiac valve defects, aneurysms and vascular prostheses are at risk of developing chronic Q-fever [3]. Laboratory diagnosis of acute Q-fever is based on serology and/or PCR. Serology, especially with the standard indirect immunofluorescence antibody assay (IFA), is prone to variation due to subjective interpretation of results [4]. IgM and IgG antibodies against phase II antigen appear about 7–15 days after the onset of illness. In the first 2 weeks, *C. burnetii* DNA can be detected in serum [5]. Persistently elevated IgG phase I antibodies (⩾1:1024) and positive PCR, generally measured 9 months after the acute Q-fever phase, are used to diagnose chronic Q-fever [6].

In the Netherlands, just before 2007, the estimated seroprevalence of antibodies against *C. burnetii* in the general population was 2.4%, which is low compared with the seroprevalence in other countries [7, 8]. From 2000 to 2006, only 5–20 cases per year were reported to the Dutch public health authorities [9]. During the epidemic in the Netherlands, from 2007 to 2010, 4026 laboratory-confirmed acute Q-fever cases were notified [10]. However, it was estimated that one Q-fever notification represents 12.6 incident infections of *C. burnetii* [11]. The human cases were mostly living close to Q-fever-affected dairy goat farms. The epidemic was finally curbed by the culling of pregnant animals on infected farms and the mandatory vaccination of goats and sheep on farms with more than 50 animals [9].

The epidemic had a huge impact on local communities and became a political issue at the local and national level. Informing the Dutch outbreak management team, professionals, decision makers and the public about the extent and course of the epidemic was based on notifications of acute Q-fever to the local and national public health authorities. The mandatory notification that exists in many countries is not only useful to detect changes in epidemiology but also facilitates source investigation and evaluation of the effect of control measures.

During an outbreak or epidemic, changes in awareness among physicians and the public occur with both changes in health-seeking behaviour and laboratory testing practices. Also, laboratory procedures might be adapted and the dynamics of the epidemic will cause profound changes in the sero-epidemiological pattern. This may influence the interpretation of serological test results, thereby affecting the validity of the prevailing notification criteria. The aim of the present study was to describe the changes in the notification criteria that were made during the Dutch Q-fever epidemic period as a result of these developments and assess how they impacted the accuracy of identifying the cases. A secondary aim was to compare the Dutch notification criteria to those from other countries in order to analyse to which extent the international notification criteria for acute Q-fever are harmonised and can be used to establish standardised criteria for Q-fever notification.

## Methods

We used published articles on (changes in) laboratory procedures related to the Dutch epidemic and a description of changes that were made to the Dutch notification criteria. An analysis of the output of a regional laboratory with notifications to the regional Public Health Service (PHS) and the national register of infectious diseases during the epidemic was conducted. Finally, we compared the international notification criteria for acute Q-fever.

A PubMed search was carried out in order to identify the articles on (changes in) laboratory procedures related to the Dutch epidemic. We identified relevant articles submitted from January 2007 to December 2015. Search terms ‘acute Q-fever’, ‘serology’, ‘complement fixation test’, ‘ELISA’, ‘IFA’, ‘seroconversion’, ‘IgG’, ‘IgM’, ‘PCR’ and ‘nucleic acid testing’ were used (Table S1). Abstracts and titles were assessed by one investigator. Articles written in English regarding the Dutch outbreak and published between 01-01-2007 and 31-12-2015 were included. Publications from countries other than the Netherlands, animal studies, epidemiological studies, clinical studies, fundamental research, case reports, reviews and articles pertaining to chronic Q-fever were excluded. We collected data on the characteristics of tests for the diagnosis of acute Q-fever and changes in laboratory procedures. Conclusions from selected articles were compared with notification criteria.

Information on changes to the Dutch notification criteria for acute Q-fever was obtained from unpublished documents archived at the National Institute for Public Health and the Environment (RIVM) and was used to describe the pivotal episodes during the epidemic that influenced the notification data.

In the Netherlands, both the microbiology laboratories and treating physicians are obliged to notify the cases of acute Q-fever to the PHS. Subsequently, the PHS verifies if the cases match the national notification criteria. Cases that fit the criteria are reported in the national register of infectious diseases. We analysed the Q-fever laboratory diagnoses notified by the laboratory of medical microbiology of the Jeroen Bosch Hospital (LMM JBZ) to the PHS Hart voor Brabant (HvB) between 01-01-2007 and 31-12-2012. Notifications up to and including 2012 were analysed in order to include the aftermath of the epidemic. The PHS HvB covers a population of about 1 million citizens in the south of the Netherlands. Reasons for not reporting cases in the national infectious disease register were analysed according to the prevailing notification criteria. Cases that failed to meet the national notification criteria were categorised in the groups: not fitting the laboratory notification criteria, onset of illness more than 90 days ago (which became an exclusion criterion in the course of the epidemic) and not fitting the clinical criteria.

In order to analyse whether the international notification criteria for acute Q-fever are harmonised, we compared the notification criteria of 2016–2017 of the European Centre for Disease Prevention and Control (ECDC), the US Centers of Disease Control and Prevention (CDC), Australia, Germany and the revised 2014 Dutch notification criteria (Table S2). Bulgaria, Cyprus, Croatia and Hungary use the EU case definition. Germany uses its own case definition. Although Q-fever is also highly prevalent in France, notification of acute Q-fever is not mandatory and thus France was not included in this study. In Belgium, Denmark, the UK and Spain, notification of acute Q-fever likewise is not mandatory [12].

## Results

The PubMed search on changes in laboratory procedures during the epidemic yielded 79 references (Table S1 and Fig. S1). Of these, 30 articles dealt with the Dutch epidemic. After the screening of titles and abstracts, nine articles were selected for full-text review [13–21]. Two review articles, two case reports, 11 articles on chronic Q-fever, four articles focusing on clinical aspects and two articles on basic pathophysiologic aspects of *C. burnetii* were considered not relevant for our study aim.

### PCR

*C. burnetii* DNA can be detected in serum by PCR up to 17 days after onset of clinical signs and symptoms. PCR has been shown to have high sensitivity (92.2–98%) and specificity (98.9–100%) [20, 21]. In case of a positive PCR combined with an IgG phase I antibody response, chronic Q-fever must be excluded [6].

### IgM phase II

After acute *C. burnetii* infection, the IgM phase II antibody response can be measured by both IFA and ELISA (sensitivity of 98% and 92%, respectively) [14, 15, 18]. For detection of an isolated IgM phase II response, IFA or ELISA have a low positive predictive value of 65% and 51%, respectively [17]. A prolonged persistence of IgM phase II antibodies was shown in antibody models [13, 16]. Moreover, in well-defined follow-up samples at 12 months, 20% of samples showed high levels of IgM phase II titres (⩾1:128) [15].

### IgG phase II

After an acute *C. burnetii* infection, the IgG phase II antibody response can be measured with IFA (sensitivity of 100%), with ELISA (sensitivity of 95%) and complement fixation test (CFT) (sensitivity of 97%). Post-infection, seroconversion intervals of 10–15 days for IgG phase II were detected [20]. A large dataset (*n* = 2321) of serological follow-up samples from acute Q-fever patients showed that after clinical presentation of the disease, the average time to a measurable antibody response for IgG phase II started at day 5 and peaked at day 18 (time to peak) [13].

### Laboratory logistics

In April 2009, the LMM JBZ added screening with ELISA IgM phase II and PCR to the routine diagnostic workflow with IFA for acute Q-fever [15, 19]. The ELISA IgM screening test was introduced as a screening tool to cope with the diagnostic demand as screening with IFA is very labour intensive [15, 19].

### Notification criteria in the Netherlands

Q-fever has been a notifiable disease in the Netherlands since 1975. In 1995, a national committee (Dutch acronym: LOI) was appointed by the Dutch government in order to realise a more structured policy on infectious disease control. Representatives of the PHS, the RIVM and the Dutch Society of Medical Microbiology are members of the committee. Changes in the notification criteria have to be endorsed by LOI and are proposed when there are important changes in epidemiology, diagnostic methods or insights of the interpretation of diagnostic tests. Prior to the start of the outbreak in 2007, the notification criteria consisted of a matching clinical presentation and a fourfold titre rise of *C. burnetii*-specific antibodies, measured in paired samples taken 2 weeks apart, or a positive IgM titre or antibodies against phase I (compatible with chronic Q-fever). Prior to 2007, the seroprevalence of *C. burnetii* antibodies in the general population was low. Thus, a single positive IgM titre, in combination with compatible clinical symptoms, was considered sufficient for diagnosis and notification as acute Q-fever. In 2008, a ‘fourfold titre increase of *C. burnetii*-specific antibodies’ was defined more specifically, being either IgG measured by IFA or CFT. Furthermore, a ‘positive IgM titre’ applied to phase II. Another change in 2008 was the addition of the possibility to notify ‘probable cases’. A probable case was defined as a matching clinical picture and a single high IgG titre or a single positive CFT. In 2008, clinical symptoms for notification were specified as fever, pneumonia or hepatitis. In 2010, ELISA and *C. burnetii* PCR on blood or respiratory material were added as diagnostic options. Furthermore, the increasing number of cases with a positive serological laboratory test but without recent clinical symptoms led to the decision to notify only cases with an onset of illness <90 days before laboratory diagnosis. Cases were notified by the LMM JBZ to the PHS HvB based on the actual laboratory case definitions followed by the PHS HvB checking the clinical data.

The results of notifications of the LMM JBZ to the PHS HvB and national notifications are depicted in Table 1. As no clinical restrictions existed in 2007 regarding national notification criteria, every positive laboratory diagnosis submitted by the LMM JBZ to the PHS HvB was notified nationally. From 2008 onwards, changes in PHS HvB data registration made extraction of the notifying laboratory possible (Table 1). The percentage of nationally notified cases decreased from 100% in 2007 and 2008 to 7.8% in 2012. The most frequent reason for not notifying nationally (58.1% (487/838)) was that the patient did not fit the nationally dictated clinical criteria. For 41.9% (350/838), the reason for not notifying nationally was that the first day of illness was more than 90 days before notification. In 2008, almost all notified cases were detected by IFA. In 2009, up to 27.3% (305/1117) of notified cases were diagnosed with PCR, compared with 4.6% (6/130) of cases in 2010 after control measures had been taken.

**Table 1.** Q-fever cases notified by LMM JBZ and nationally notified by the MHS 2008 up to 2013. Non-notified cases are specified by test and reason

Year			2008		2009		2010		2011		2012		Total	
			*n*	*%*	*n*	*%*	*n*	*%*	*n*	*%*	*n*	*%*	*n*	*%*
Q-fever testing requests JBZ			5142		13 120		16 469		8028		6277			
**MHS registered Q-fever-positive cases from JBZ**			**643**	***100***	**1365**	***100***	**512**	***100***	**137**	***100***	**90**	***100***	**3147**	***100***
** Nationally notified by MHS**			**643**	***100***	**1117**	***81.8***	**130**	***2.4***	**12**	***8.8***	**7**	***7.8***	**2133**	***67.8***
	Test	Elisa	0		34		0		0		0		**34**	
		CBR	1		2		0		0		0		**3**	
		IFA	642		480		124		12		7		**1265**	
		IFA + PCR	0		18		0		0		0		**18**	
		PCR	0		305		6		0		0		**311**	
** Cases not fitting national notification criteria**			**0**		**248**	***18.2***	**382**	***746***	**125**	***91.2***	**83**	***92.2***	**838**	***33.2***
	Test	Elisa			4		2		0		0		**6**	
		CBR			0		0		0		0		**0**	
		IFA			230		359		124		83		**796**	
		IFA + PCR			0		1		0		0		**1**	
		PCR			7		5		1		0		**13**	
		combinations/unknown			7		15		0		9		**31**	
** Reasons not notified**^a^														
Not fitting laboratory test criteria					1		0		0		0		**1**	
First day of illness >90 days ago					na		162		109		79		**350**	
Not fitting clinical criteria					247		220		16		4		**487**	

Bold numbers represent total numbers Italics represent percentages.

a Only one reason was presented in this table although all could apply to the same person. The cases were assessed in the order presented in this table.

**Fig. 1.**
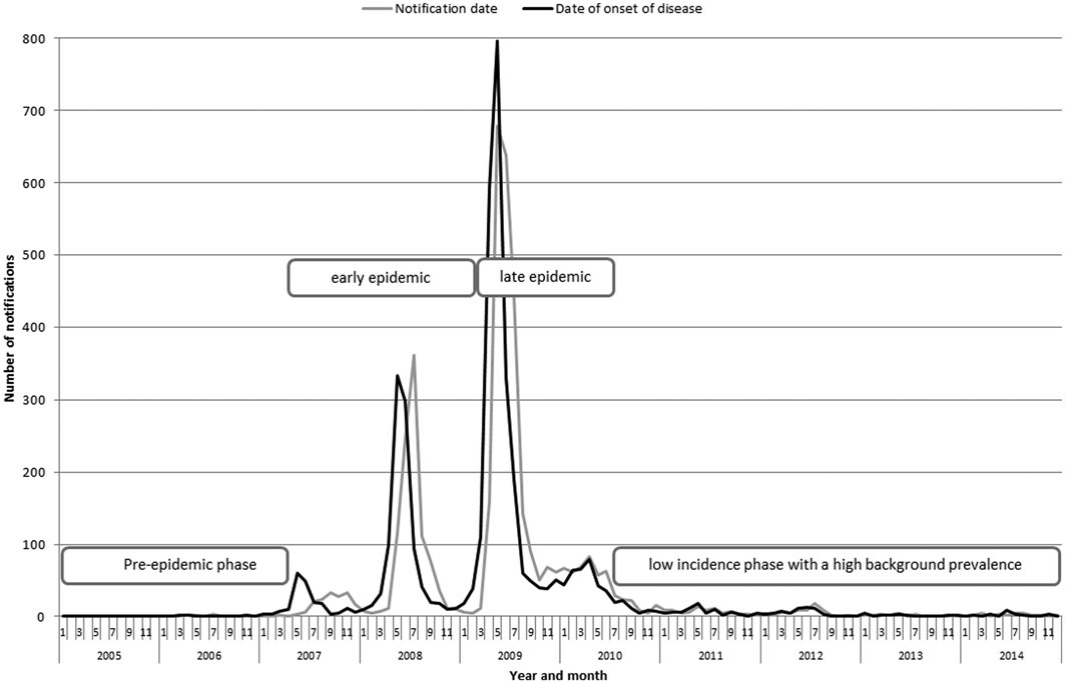
Number of notifications of acute Q fever registered in the national notification database by year and month.

Cases registered in the national register of infectious diseases with dates of onset of disease and notification are shown in Figure 1. In the epidemic, four relevant periods can be identified: (1) the steady-state pre-epidemic phase (seroprevalence 2.4%) [8]; (2) the early epidemic phase with high incidence rates in combination with a low background seroprevalence (9–12.2%) [22, 23]; (3) the high incidence late epidemic phase with an increasing background seroprevalence and (4) the low incidence post-epidemic phase with a high background seroprevalence (6.1–33.8%) [24, 25].

### International notification criteria

We analysed the international notification criteria for acute Q-fever (Tables S2 and S3). A positive *C. burnetii* PCR in combination with the clinical criteria is unanimously accepted as sufficient for notification of acute Q-fever [26–30]. Fever, signs of hepatitis and/or pneumonia are included in the criteria of the CDC, the ECDC, Germany and the Netherlands [27–30]. CDC additionally mentions severe retrobulbar headache and elevated liver enzymes [27]. The Australian notification criteria do not specify the symptoms compatible with acute Q-fever [26]. The interpretation of the presence of IgM or IgG antibodies differs internationally. In Australia, detection of a specific IgM antibody response, in the absence of recent vaccination, is suggestive evidence of acute Q-fever. Seroconversion or a significant increase in phase II antigens in paired sera tested parallel, in the absence of recent vaccination, is definitive evidence [26]. In the EU case definition, a *C. burnetii*-specific phase II response is interpreted as an acute Q-fever infection [28]. In the CDC criteria, confirmative criteria consist of serological evidence of a fourfold change in IgG phase II between paired sera. Elevated phase II IgG or IgM antibodies, tested by ELISA or latex agglutination, or a single IgG titre of phase II antigen of ⩾1:128 tested by IFA are supportive evidence [27]. The German criteria mention a significant change in IgM or IgG between two samples and do not specify the amount of increase in antibody levels [30]. In the Dutch criteria, seroconversion or a fourfold rise in IgG antibody titre in paired sera tested by IFA, ELISA or CFT or the presence of IgM phase II antibody were confirmative criteria for the notification of acute Q-fever. Cases with indications of chronic Q-fever are not notifiable [29].

## Discussion

We studied the changes in the notification criteria during the Q-fever epidemic that occurred in the Netherlands from 2007 to 2010. We identified four pivotal periods that require specific notification rules to acquire accurate and useful notification data: (1) the steady-state pre-epidemic phase; (2) the early epidemic phase with high disease incidence rates in combination with a low background seroprevalence; (3) the high disease incidence late epidemic phase with an increasing background seroprevalence and (4) the low disease incidence post-epidemic phase with a high background seroprevalence.

Each of these four periods has specific sero-epidemiological features, affecting the reliability of notification criteria. This is further confounded by increasing awareness of the disease as the epidemic develops, with changes in health-seeking behaviour, more requests for laboratory testing and changes in laboratory procedures.

### The steady-state pre-epidemic phase

The seroprevalence of antibodies against *C. burnetii* just before the outbreak in 2007 was low in the Netherlands, in comparison to other European countries [7, 8]. This was based on sera collected from a large (*n* = 5654) nationally representative sample of the general Dutch population and revealed a seroprevalence of 2.4% during February 2006 to May 2007, before the first outbreak in June 2007 based on IgG II in ELISA ⩾20 U/ml, and IgG II ⩾1:32 in IFA on a subset [8]. There was little awareness of the disease and isolated cases were usually identified by chance, because of unexplained clinical presentation and persistence of astute clinicians to confirm a diagnosis. However, there is evidence that there was already an increased zoonotic transmission of *C. burnetii* in the 2 years before the epidemic became apparent [31]. For specific laboratory diagnosis of Q-fever, serum had to be sent to the national reference laboratory (RIVM), which performed IFA screening, followed by confirmation of IgG and IgM phase I and II with CFT. This resulted in a turn-around time of about 4 weeks. During this period, standard notification rules were used that were defined prior to 1994. The exact year of definition of these criteria is not documented.

### The high incidence early epidemic phase with a low background exposure

In spring 2007, an increasing number of acute Q-fever cases presenting with pneumonia were diagnosed. From September 2007 onwards, there was an increasing demand for laboratory testing for Q-fever. In order to speed up the turn-around time, the LMM JBZ implemented the ELISA IgM screening test resulting in faster identification of new cases [18]. Cases notified in 2007 had usually been diagnosed in retrospect and positive serology could not be interpreted consistently as different diagnostic tests, such as CFT and IFA, were used.

During the epidemic in the Netherlands, no nationwide seroprevalence surveys were conducted, but studies in the Q-fever-affected areas in the south of the country indicated a considerable increase in seroprevalence. Among 2004 pregnant women during the period of 2007–2009, the seroprevalence was 9.0%, and among 543 blood donors in 2009, it was 12.2% based on IgG II ⩾1:64 in IFA [22, 23].

### The high incidence late epidemic phase with a high background exposure

During the three seasonal outbreaks from 2007 to 2009 (Fig. 1), there was a sharp increase in incidence, both in the clinical and subclinical presentation. Therefore, in April 2009, the LMM JBZ implemented initial screening with ELISA IgM phase II and added PCR to the routine diagnostic workflow for acute Q-fever [15, 19]. The cheaper and less labour-intensive ELISA IgM screening test was introduced to cope with the diagnostic demand [15, 19]. In this period, 27.3% (305/1117) of notified cases were diagnosed with PCR. This reflects the awareness among the general public and physicians with adapted laboratory logistics leading to the early detection of Q-fever. Although PCR on serum had not yet been incorporated in the national notification criteria, PCR-positive Q-fever cases were notified nationally by the PHS. A large portion of the population had been infected early during the epidemic, often without clinical symptoms. Routine serology often revealed a positive IgG/IgM result, though cases were not infected recently. On top of this, the inter-current influenza A (H1N1) pdm09 pandemic in the second half of 2009 probably led to additional Q-fever notifications, because many patients with influenza-like illness also tested positive for antibodies against *C. burnetii.*

### The low incidence phase with a high background prevalence

The post-epidemic phase was reached after extensive control measures were taken, in particular the culling of goats and sheep on infected farms and the mandatory mass vaccination of goats and sheep. Several years after the epidemic, in 2014–2015, seroprevalence had declined to 6.1% among 2296 people from the general adult population in parts of the provinces Noord Brabant and Limburg based on IgG II in ELISA ⩾20 U/ml [24]. Where in 2009, 27.3% (305/1117) of notified cases were diagnosed with PCR, this rate dropped to 4.6% (6/310) in 2010. Although the demand for diagnostic testing of Q-fever in the community continued, the proportion of samples that tested positive for PCR decreased. At this time, laboratory notifications to the regional PHS were mainly based on the presence of IgM antibodies, reflecting past infections rather than acute infections. As the patient's anamnesis was often negative for fever, pneumonia or hepatitis, the PHS did not enter these laboratory notifications into the national infectious disease register. Based on the reduced number of PCR-positive cases, it was assumed that the risk of exposure was truly reduced, confirming the efficacy of control measures.

Until 2011, the Dutch notification criteria regarding IgM phase II antibodies remained unchanged. The detection of IgM phase II antibodies was considered as evidence of acute Q-fever, despite the altered epidemiology and implementation of IFA (April 2009), ELISA IgM phase II (June 2009) and PCR (April 2010). Jaramillo-Gutierrez *et al*. retrospectively analysed how unchanged notification criteria led to over-reporting of cases to the PHS in the post-epidemic years, caused by the increasing seroprevalence in the population. Over the 3-year period, the incidence of the confirmed cases by the PHS significantly decreased [15, 32]. Based on these results, the authors recommended omitting IgM phase II-positive sera in the notification criteria. In January 2015, the Dutch notification criteria were revised to the extent that a positive IgM has to be confirmed either by seroconversion of IgG or a positive PCR result in order to be notified as an acute Q-fever case. Without confirmation, the case is notified as probable Q-fever.

The interpretation of IgM phase II differs significantly internationally. As the sero-epidemiology of Q-fever also differs by country, this is not unexpected. In an immunologically naïve population, detection of IgM II antibodies can be a useful indicator of acute Q-fever. This is less the case in endemic areas or after an epidemic period.

ELISA and IFA measuring IgG phase II can detect seroconversion 10–15 days post-infection [33]. However, due to a rapid time to peak a fourfold rise in titre is rarely measured in paired serum samples [13, 16, 34–36]. Seroconversion and/or a significant change in IgG phase II antibody titre are included in the notification criteria of Australia and Germany. The US CDC and Dutch notification criteria include seroconversion and/or a fourfold change in IgG phase ll.

A positive PCR is unanimously accepted in international notification criteria as an inclusion criterion for notification of acute Q-fever when combined with clinical criteria (Table S2). However, this requires sampling of serum within the first 2 weeks after onset of illness and the exclusion of chronic Q-fever disease.

Clinical criteria (fever, pneumonia and/or hepatitis) were very important in combination with the laboratory results to measure the effect of the control measures and to define the tail of the epidemic curve. During the epidemic, clinical symptoms did not always discriminate between acute Q-fever and other infectious diseases, which was especially noticeable during the concurrent influenza pandemic in 2009.

### General conclusion

During this epidemic, changes in health-seeking behaviour and higher awareness among health professionals resulted in an increasing workload. This prompted changes in laboratory procedures. Screening with ELISA IgM phase II and PCR was implemented. In the steady-state pre-epidemic phase, with a low background seroprevalence, the presence of IgM antibodies against phase II of *C. burnetii* in a single serum sample was considered sufficient for diagnosis and notification as an acute Q-fever case.

However, in the current post-epidemic phase with a high background seroprevalence in the Netherlands, the presence of IgM phase II antibodies can falsely be interpreted as acute Q-fever. A positive IgM in combination with clinical criteria may still be suggestive of acute Q-fever, but has to be confirmed either by seroconversion of IgG or a positive PCR result. From 2009 onwards, nationally notified acute Q-fever cases were more often diagnosed by PCR and the percentage of positive serological laboratory tests that were notified nationally decreased.

Ideally, laboratory procedures and clinical case definitions as well as national notification criteria should not change during an outbreak. However, this is not realistic as each epidemic phase is characterised by a specific sero-epidemiological profile. Due to the long persistence of IgM phase II, this especially has implications for the interpretation of the presence of IgM II antibodies.

As the sero-epidemiology of Q-fever differs per country, the interpretation of the presence of IgM phase II antibodies differs significantly internationally. Background seroprevalence has to be taken into account in order to reliably interpret notification data on acute Q-fever. In the European Union region, acute Q-fever is a notifiable disease but many countries do not have a notification system for acute Q-fever or do not comply with the European Commission case definition. The present study shows that because of geographic differences and temporal changes in sero-epidemiology, full harmonisation of notification criteria between countries and within countries might not be feasible.

## Supplementary material

For supplementary material accompanying this paper visit https://doi.org/10.1017/S0950268819000736. click here to view supplementary material
