# Supplementary material for: Notification data and criteria during a large Q-fever epidemic reassessed
Source: Epidemiol Infect. 2019 May 9;147:e191. doi: 10.1017/S0950268819000736 (PMC6518550; doi:10.1017/S0950268819000736)
Supplement: Supplementary file 1 [file S0950268819000736sup001.docx]

**Supplementary Figure 1: Flowchart of identified articles**

79 articles on PubMed

Articles not concerning Dutch epidemic:

49 articles excluded

30 articles identified as potentially relevant

21 articles excluded

2 review articles

2 case reports

11 articles on chronic Q-fever or follow up/detection of chronic Q-fever

4 articles focusing on clinical aspects

2 articles on basal pathophysiologic aspects of Q-fever

9 articles included

**Supplementary Table 1: Search strategy used in Pubmed**

| ((((((((("acute q fever"[All Fields] AND ("2007/01/01"[PDAT] : "2015/12/31"[PDAT]) AND English[lang]) AND "serology"[All Fields] AND ("2007/01/01"[PDAT] : "2015/12/31"[PDAT]) AND English[lang]) OR (("acute q fever"[All Fields] AND ("2007/01/01"[PDAT] : "2015/12/31"[PDAT]) AND English[lang]) AND "pcr"[All Fields] AND ("2007/01/01"[PDAT] : "2015/12/31"[PDAT]) AND English[lang])) OR (("acute q fever"[All Fields] AND ("2007/01/01"[PDAT] : "2015/12/31"[PDAT]) AND English[lang]) AND "complement fixation test"[All Fields] AND ("2007/01/01"[PDAT] : "2015/12/31"[PDAT]) AND English[lang])) OR (("acute q fever"[All Fields] AND ("2007/01/01"[PDAT] : "2015/12/31"[PDAT]) AND English[lang]) AND "IFA"[All Fields] AND ("2007/01/01"[PDAT] : "2015/12/31"[PDAT]) AND English[lang])) OR (("acute q fever"[All Fields] AND ("2007/01/01"[PDAT] : "2015/12/31"[PDAT]) AND English[lang]) AND "ELISA"[All Fields] AND ("2007/01/01"[PDAT] : "2015/12/31"[PDAT]) AND English[lang])) OR (("acute q fever"[All Fields] AND ("2007/01/01"[PDAT] : "2015/12/31"[PDAT]) AND English[lang]) AND "igm"[All Fields] AND ("2007/01/01"[PDAT] : "2015/12/31"[PDAT]) AND English[lang])) OR (("acute q fever"[All Fields] AND ("2007/01/01"[PDAT] : "2015/12/31"[PDAT]) AND English[lang]) AND "igg"[All Fields] AND ("2007/01/01"[PDAT] : "2015/12/31"[PDAT]) AND English[lang])) OR (("acute q fever"[All Fields] AND ("2007/01/01"[PDAT] : "2015/12/31"[PDAT]) AND English[lang]) AND "nucleic acid testing"[All Fields] AND ("2007/01/01"[PDAT] : "2015/12/31"[PDAT]) AND English[lang])) OR (("acute q fever"[All Fields] AND ("2007/01/01"[PDAT] : "2015/12/31"[PDAT]) AND English[lang]) AND "seroconversion"[All Fields] AND ("2007/01/01"[PDAT] : "2015/12/31"[PDAT]) AND English[lang]) AND (("2007/01/01"[PDAT] : "2015/12/31"[PDAT]) AND English[lang]) |
| --- |

**Supplementary Table 2: Comparison of national and international notification criteria**

| **References:**  Australian notification criteria. Ref: <http://www.health.gov.au/internet/main/publishing.nsf/Content/cda-surveil-nndss-casedefs-cd_qfev.htm>  CDC surveillance case definition and case classification for acute Q-fever. Ref: <https://wwwn.cdc.gov/nndss/conditions/q-fever/case-definition/2009/>  Harmonised case definition of human Q-fever under EU legislation. Ref: <http://eur-lex.europa.eu/legal-content/EN/TXT/PDF/?uri=CELEX:32012D0506&qid=1428573336660&from=EN#page=24>  German notification criteria. Ref: <https://www.rki.de/DE/Content/Infekt/IfSG/Falldefinition/Downloads/Falldefinitionen_des_RKI.pdf?__blob=publicationFile>  Dutch notification criteria. Ref: <http://www.rivm.nl/Documenten_en_publicaties/Professioneel_Praktisch/Richtlijnen/Infectieziekten/LCI_richtlijnen/LCI_richtlijn_Q_koorts> | | | | | |
| --- | --- | --- | --- | --- | --- |
|  | **Australia** | **CDC** | **ECDC** | **Germany** | **The Netherlands** |
| **Clinical criteria** | A clinically compatible disease | **Fever and one or more of the following:**  Rigors, severe retrobulbar headache, acute hepatitis, pneumonia, or elevated liver enzymes | **Any person with at least one of the following three:**  Fever, pneumonia or  Hepatitis | **Any person with at least one of the following four:**  General signs of illness, fever, signs of hepatitis or pneumonia  OR  Disease related death | **Any person with at least one of the following three:**  Fever, pneumonia or hepatitis |
| **Laboratory criteria** | **Laboratory confirmed:**  Detection of *C. burnetii* by nucleic acid testing  OR  Seroconversion or significant increase in antibody level to Phase ll antigens in paired sera tested in parallel in absence of recent Q-fever vaccination  OR  Detection of *C. burnetii* by culture  **Laboratory suggestive:**  Detection of specific IgM in the absence of recent Q-fever vaccination | **Laboratory confirmed:**  Serological evidence of a fourfold change in IgG antibody titer to *C. burnetii* phase ll antigen by IFA between paired serum samples  OR  Detection of *C. burnetii* DNA in a clinical specimen via amplification of a specific target by PCR assay  OR  Demonstration of *C. burnetii* in a clinical specimen by IHC methods  OR  Isolation of *C. burnetii* from a clinical specimen by culture  **Laboratory supportive:**  Has a single supportive IFA IgG titer of ≥1:128 to *C. burnetii* phase ll antigen by IFA (phase l titers may be elevated as well)  Has serological evidence of elevated phase ll IgG or IgM antibody reactive with *C. burnetii* antigen by ELISA, dot-ELISA, or latex agglutination | **At least one of the following three:**  Isolation of *C. burnetii* from a clinical specimen  Detection of *C. burnetii* nucleic acid in a clinical specimen  *C. burnetii*-specific antibody response (IgG or IgM phase ll) | **At least one of the following four:**  Isolation of *C. burnetii* from a clinical specimen  Detection of *C. burnetii* nucleic acid in a clinical specimen  IgM: Significant change between two samples or one individual sample significant elevated  IgG: Significant change between two samples | **At least one of the following three:**  Seroconversion or a fourfold change in immunoglobulin G (IgG)-specific antibody titer to *C. burnetii* between paired sera (serum of acute phase and serum collected at least 2 weeks later) detected by IFA or CFT  OR  Detection of IgM antibody titer to *C. burnetii* phase ll antigen  OR  Detection of *C. burnetii* DNA in a clinical specimen by PCR or isolation of *C. burnetii* from a clinical specimen by culture (not notifiable in case of chronic Q-fever) |
| **Epidemiological criteria** |  |  | **At least one of the following two epidemic links:**  Exposure to a common source  Animal-to-human transmission  Case classification | **Epidemiological confirmation defined by one of the following (considering the incubation period):**  Epidemiological link with a laboratory-confirmed case through exposure to a common source.  Exposure to a laboratory-confirmed infected animal, its excretions or its afterbirth |  |
| **Case classification** | **Confirmed case:**  A confirmed case requires either:   1. Laboratory definitive evidence   OR   1. Laboratory suggestive evidence AND clinical evidence. | **Confirmed case:**  A laboratory confirmed case that either meets clinical criteria or is epidemiologically linked to a lab confirmed case  **Probable case:**  A clinically compatible case of acute illness (meets clinical evidence criteria for acute Q-fever illness) that has laboratory supportive results for past or present acute disease (antibody to Phase II antigen) but is not laboratory confirmed | **Confirmed case:**  Any person meeting the clinical and the laboratory criteria  **Probable case:**  Any person meeting the clinical criteria and with an epidemiological link | **Clinical and epidemiological confirmed case:**  Any person meeting the clinical without laboratory evidence but with an epidemiological link  **Clinical and laboratory confirmed case:**  Any person meeting the clinical with laboratory evidence  **Laboratory evidence of infection without clinical criteria:**  Any person not meeting the clinical criteria with laboratory evidence.  **Laboratory evidence of infection with unknown clinical criteria.** | **Confirmed case:**  Any person meeting the clinical criteria and has one of the following laboratory criteria:  Seroconversion or a fourfold change in immunoglobulin G (IgG)-specific antibody titer to *C. burnetii* between paired sera  OR  Detection of *C. burnetii* DNA in a clinical specimen by PCR or isolation of *C. burnetii* from a clinical specimen by culture  **Probable case:**  Any person meeting the clinical criteria and the laboratory criteria |
| **Note** | Only confirmed cases are notifiable. Confirmed cases require either laboratory definitive evidence, or laboratory suggestive evidence together with clinical evidence. | CDC suggests one taken during the first week of illness and a second 3-6 weeks later, antibody titers to phase I antigen may be elevated or rise as well  For acute testing, CDC uses in-house IFA IgG testing (cutoff of ≥1:128), preferring simultaneous testing of paired specimens, and does not use IgM results for routine diagnostic testing |  | IgM antibodies can persist for a long period of time  Antibodies can be detected against phase-l or phase-ll antigen | Probable cases are notifiable  Chronic Q-fever and Q-fever fatigue syndrome are not notifiable |

**Supplementary Table 3: Notification criteria for Q-fever in European countries: Annual epidemiological report 2014**

| **Reference:** <http://ecdc.europa.eu/en/publications/Publications/emerging-vector-borne-diseases_annual-epidemiological-report-2014.pdf> | | |
| --- | --- | --- |
| **Country** | **Compulsory (cp) / Voluntary (V)** | **Case definition used** |
| Belgium | V | Not specified/ unknown |
| Bulgaria | Cp | EU-2008 |
| Cyprus | Cp | EU-2008 |
| Czech Republic | Cp | EU-2008 |
| Denmark | - | - |
| Estonia | Cp | EU case definition (legacy/deprecated) |
| Finland | Cp | Not specified/unknown |
| France | V | Other |
| Germany | Cp | Other |
| Greece | Cp | EU-2008 |
| Hungary | Cp | EU-2002 |
| Iceland | Cp | EU-2008 |
| Ireland | Cp | EU-2008 |
| Latvia | Cp | EU-2012 |
| Lithuania | Cp | EU-2008 |
| Luxembourg | Cp | EU-2002 |
| Malta | Cp | EU-2008 |
| Netherlands | Cp | EU-2008 |
| Norway | Cp | EU-2012 |
| Poland | Cp | EU-2008 |
| Portugal | Cp | EU-2008 |
| Romania | Cp | EU-2008 |
| Slovakia | Cp | EU-2012 |
| Slovenia | Cp | EU-2008 |
| Spain | V | EU-2008 |
| Sweden | Cp | EU-2012 |
| United Kingdom | V | EU-2012 |
